# The current state of pediatric gastroenterology in under-resourced nations

**DOI:** 10.1097/MS9.0000000000003141

**Published:** 2025-03-07

**Authors:** Medha Sridhar Rao, Aditya Gaur, Hareesha Rishab Bharadwaj, Shahzeb Imran, Joecelyn Kirani Tan, Saad Abbas, Muhtasim Fuad, Shadi Abuhashem, Muhammad Hamza Shah, Priyal Dalal, Abdulrahman Nasir Al Khatib, Khabab Abbasher Hussien Mohamed Ahmed

**Affiliations:** a School of Medicine, Dentistry and Biomedical Sciences, Queen’s University Belfast, Belfast, United Kingdom; b Yeovil District Hospital, Somerset NHS Foundation Trust, Higher Kingston, Yeovil, United Kingdom; c Faculty of Biology Medicine and Health, The University of Manchester, Manchester, United Kingdom; d Al-Quds University, Jerusalem, West Bank, Palestine; e School of Medicine and Dentistry, University of Central Lancashire, Preston, United Kingdom; f Faculty of Medicine, The University of Jordan, Amman, Jordan; g Faculty of Medicine, University of Khartoum, Khartoum, Sudan

**Keywords:** celiac disease (ceD), healthcare access, inflammatory bowel disease (IBD), low- and middle-income countries (lMICs), paediatric gastrointestinal diseases

## Abstract

**Background:**

Pediatric gastroenterology (GI) care in low- and middle-income countries (LMICs) faces substantial challenges due to limited healthcare infrastructure, inadequate resources, and a shortage of specialized healthcare professionals. These challenges lead to delayed diagnoses and treatment, exacerbating the morbidity and mortality associated with pediatric GI diseases, which include both infectious conditions like diarrhea and chronic conditions such as inflammatory bowel disease (IBD) and liver diseases.

**Aim:**

The aim of this review is to examine the current state of pediatric GI care in LMICs, identify the key challenges these regions face, and propose strategies to improve healthcare outcomes for children affected by GI disorders.

**Methods:**

This review synthesizes existing literature from a range of LMICs, analyzing factors such as the economic burden of healthcare, barriers to access, the availability of diagnostic and therapeutic services, and the state of pediatric hepatology and endoscopy. Studies included in the review were sourced from countries in sub-Saharan Africa, South Asia, and other LMIC regions, focusing on pediatric GI disorders and healthcare delivery.

**Results:**

Economic burden: Families in LMICs face significant economic barriers in accessing pediatric GI care, with treatment costs often exceeding household income, especially in private healthcare settings. Healthcare access: Limited access to healthcare facilities, especially in rural areas, coupled with the shortage of trained pediatric gastroenterologists and necessary medical equipment, leads to delayed diagnoses and inadequate care for conditions like *Helicobacter pylori* infections and chronic liver diseases. Sanitation and infectious diseases: Poor sanitation and lack of access to clean water contribute to the high prevalence of diarrheal diseases, which can be reduced through better hygiene practices and improved infrastructure. Training gaps: The shortage of trained healthcare workers, particularly pediatric specialists, hinders effective care delivery, with healthcare workers often overburdened due to workforce migration and low salaries. Hepatology and endoscopy: Pediatric hepatology, especially in the context of viral hepatitis, and the availability of pediatric GI endoscopy are severely limited in LMICs, further complicating the management of liver diseases and GI conditions in children.

**Conclusion:**

Improving pediatric GI care in LMICs requires addressing systemic challenges such as inadequate healthcare infrastructure, limited financial resources, and a shortage of trained professionals. Prevention strategies like vaccination, sanitation improvements, and public health education campaigns are crucial for reducing the prevalence of pediatric GI diseases. In addition, enhancing access to specialized training, healthcare services, and diagnostic tools will improve outcomes for children in resource-limited settings. Continued international collaboration and investment in local healthcare systems are essential for creating sustainable solutions and bridging the gap in pediatric GI care.

## Introduction

Non-infectious pediatric GI conditions include inflammatory bowel diseases such as Crohn’s disease and ulcerative colitis (UC) and other conditions including celiac disease. These conditions often have a strong genetic and autoimmune component. The genes commonly associated with these conditions are HLA-DQ2, HLA-DQ, IL18RAP, PTPN2, TAGAP, and PUS10^[1]^. The inflammation caused by these conditions can manifest as changes in stools, per rectal bleeding, ulcers in the GI tract, and improper absorption of nutrients from food. Crohn’s and UC are normally pharmacologically controlled, and CeD is treated with a strict gluten-free diet.

**HIGHLIGHTS**

- The prevalence of pediatric gastrointestinal (GI) diseases, including inflammatory bowel disease (IBD), celiac disease (CeD), and infections like rotavirus and hepatitis, is increasing in low- and middle-income countries (LMICs) due to urbanisation and inadequate healthcare infrastructure.
- Vaccines for GI infections, such as rotavirus, hepatitis A virus (HAV), and hepatitis B virus (HBV), are essential for reducing disease burden in LMICs but are limited by affordability and logistical challenges.
- Limited pediatric endoscopy and hepatology services in LMICs, driven by financial constraints and a shortage of trained professionals, exacerbate the management of GI disorders.
- Improving sanitation, hygiene education, and access to clean water are key to preventing GI infections in LMICs.
- Strengthening healthcare systems through policy development, targeted training, and local capacity-building is crucial for addressing barriers to pediatric GI care in LMICs.

Infectious pediatric GI conditions include gastritis and hepatitis caused by various pathogens including viruses, e.g. norovirus, bacteria, e.g. *Campylobacter jejuni*, and parasites, e.g. *Plasmodium falciparum*^[2]^. These conditions are often transmitted through poor sanitation, water, and food quality causing GI symptoms such as diarrhea, which leads to dehydration and, consequently, pediatric death. The treatment of these conditions is normally pharmacological such as antibiotics and fluids; preventative measures are fundamental in managing infectious conditions by reducing their spread among children and the wider population.

The World Bank^[3]^ classifies countries as being high-, middle-, or low-income, and this review utilized this classification to obtain data relating to pediatric GI care in these countries.

The state of pediatric gastroenterology in low- and middle-income countries (LMICs) faces significant challenges, with pediatric GI disorders like inflammatory bowel disease (IBD), celiac disease (CeD), and chronic liver diseases posing serious health concerns. IBD, including Crohn’s disease (CD) and ulcerative colitis (UC), is increasingly recognized in LMICs, though it remains less understood compared to high-income countries (HICs)^[4]^. CeD, an autoimmune disorder triggered by gluten, also affects children globally, but its diagnosis is often delayed in LMICs due to limited awareness and resources. Chronic liver diseases, particularly viral hepatitis and non-alcoholic fatty liver disease (NAFLD), burden healthcare systems in sub-Saharan Africa and parts of Asia.

Care delivery is hindered by inadequate healthcare infrastructure, financial constraints, and a shortage of trained professionals^[5]^. Many hospitals in LMICs struggle with overcrowding, lack of essential equipment, and limited access to advanced therapies and provider training^[6]^. Cultural and social barriers, including stigma and limited health education, further complicate diagnosis and treatment. Addressing these core issues is essential for advancing pediatric gastroenterology services and outcomes in LMICs. There is a large gap in the literature about pediatric gastrointestinal diseases and disorders in developing countries as most of the data are from developed countries. Therefore, this review explores the epidemiology of key GI conditions, the challenges limiting pediatric gastroenterology’s development, and strategies to enhance care and infrastructure.

## Methodology

This narrative review systematically gathered and assessed the literature on pediatric gastroenterology in LMICs. The search covered multiple databases – PubMed, Google Scholar, Cochrane Library, EMBASE, CINAHL, SCOPUS, and Scielo – spanning studies from their inception to 16 August 2024. Inclusion criteria encompassed diverse study designs, including observational, case-control, cohort, and randomized controlled trials, to ensure thorough evidence coverage. The articles were quality assessed using the SANRA framework for narrative reviews. The summary tables of articles used in this paper are available at the end of this article.

LMICs were identified using World Bank classification, and search terms included “pediatric gastroenterology,” “infectious gastroenterological diseases,” “diarrhoea,” and “malnutrition,” combined with geographical identifiers for LMICs. A manual review of references from recent reviews was also conducted. Stand-alone abstracts, unpublished studies, and trial protocols were excluded to maintain study quality and relevance.

### Current burden and management of pediatric GI disease in LMICs

**a) GI inflammatory diseases**

**Inflammatory bowel diseases**

Pediatric GI inflammatory diseases, such as IBD – including ulcerative colitis (UC) and Crohn’s disease (CD) – and celiac disease (CeD), are rising public health concerns in LMICs. Traditionally more prevalent in high-income countries (HICs), these conditions now show increasing incidence and prevalence in developing regions, posing significant physical, psychological, and financial burdens on affected children and their families. Pediatric IBD, driven by factors like urbanization and lifestyle changes, presents unique challenges in LMICs, where limited data complicate understanding of its full impact. Globally, an estimated 7 million people live with IBD, with recent studies indicating a notable rise in pediatric cases in the Asia-Pacific region and incidence rates nearing HIC levels in countries like India and China^[7–10]^. LMICs face severe consequences, as IBD patients have a threefold increase in mortality risk^[11]^ but lack adequate diagnostic tools like endoscopy and access to advanced treatments such as biologics^[12]^.

The complexity of managing IBD in LMICs is exacerbated by high medication costs, limited resources for specialized diets, and serious complications like toxic megacolon, which often necessitate surgeries that may be inaccessible due to financial and infrastructure constraints^[13]^. In LMICs, CD diagnosis commonly relies on endoscopy, pathology, and radiology, and when medical treatments fail, surgeries like bowel resections are often necessary. However, these procedures are challenging to consistently provide results due to resource limitations, resulting in poorer outcomes for pediatric patients^[14,15]^. These challenges underscore an urgent need to improve healthcare infrastructure, diagnostic capacity, and access to therapies to address the rising burden of pediatric GI diseases in LMICs^[16–19]^ (see Table 1).

**Table 1:** Summary of IBD-focused studies.

Study Author	Country	Date	Major Qualitative Findings
Huang JG et al.	Asia-Pacific	May 2022	Pediatric IBD diagnoses are more common in early adolescence, with geographical variability in incidence and symptoms such as abdominal pain, diarrhea, and weight loss. Treatment approaches and diagnostic delays reflect disparities in resources, emphasizing the need for standardized protocols.
Kuenzig ME et al.	LMICs	Jan 2022	The highest incidence and prevalence of pediatric-onset IBD are in Northern Europe and North America. 84% of studies reported significant incidence increases over time, and all studies assessing prevalence noted significant rises. Data on very early-onset IBD (VEO-IBD) remain limited.
Srivastava A et al.	India	Aug 2020	Pediatric IBD cases are rising in India, with Crohn’s disease more prevalent than ulcerative colitis. Common symptoms include abdominal pain, diarrhea, and growth retardation. Awareness and early diagnosis are crucial to manage the increasing cases effectively.
Odeghe OF et al.	Nigeria	Apr 2020	IBD is rare in Black African children due to diagnostic challenges in resource-constrained environments. Symptoms include chronic diarrhea, abdominal pain, and weight loss. Awareness, improved diagnostic facilities, and training are essential to better manage pediatric IBD.

**Celiac Disease**

CeD is an autoimmune inflammatory condition triggered when an individual consumes gluten or molecules similar to gluten. It is strongly associated with HLA-DQ gene mutations, and environmental factors such as pediatric gastrointestinal infections and the early introduction of gluten into a child’s diet have also been suggested as contributors. CeD leads to inflammation of the villi in the small bowel, and repeated gluten consumption can cause villous atrophy, resulting in a decreased ability to absorb essential nutrients from food, such as iron, folate, and vitamin D. CeD is increasingly being recognized in LMICs, contrary to its historical association with HICs. While the global incidence of CeD in children is estimated at 21.3 per 100,000 person-years, recent studies reveal rising rates in countries like India, Vietnam, and China, where up to 2% of children may be affected^[20–22]^. The true prevalence remains under-recognized due to limited awareness and diagnostic capacity, with healthcare providers in LMICs often overlooking CeD, associating it mainly with HICs^[23,24]^. Managing CeD is particularly difficult in resource-poor settings where gluten-free options are scarce, and gluten cross-contamination is common. Diagnostic limitations, such as restricted access to genetic testing and endoscopy, further delay treatment, leading to severe outcomes like malnutrition, growth failure, and increased mortality in untreated children^[25–28]^. CeD could be on the rise due to an increasing adoption of a gluten-based Western-style diet into the diets of LMIC populations especially in countries where carbohydrates like rice are traditionally more common, e.g. China^[29]^. Additionally, CeD cases may be silent, resulting in patients who have the condition or gene being asymptomatic; these patients can live their lives without being aware of the condition, and hence, prevalence and the actual number of cases may actually be higher than recorded^[29]^. Addressing these issues will require improved diagnostic infrastructure, greater public awareness, and better strategies for managing gluten exposure in LMIC settings (see Table 2).

**Table 2:** Summary of CD-focused studies.

Study Author	Country	Date	Major Qualitative Findings
Kalle Kurppa et al.	LMICs	Jan 2024	Pediatric IBD cases are rising in India, with Crohn’s disease more prevalent than ulcerative colitis. Common symptoms include abdominal pain, diarrhea, and growth retardation. Awareness and early diagnosis are crucial to manage the increasing cases effectively.
Gupta R et al	India	2009	Limited awareness and diagnostic facilities contribute to the underdiagnosis of celiac disease in India. The Indian Task Force for Celiac Disease emphasizes the need for improved awareness and research.
Chen CY et al.	China	Jul 2019	Despite increasing prevalence, celiac disease remains under-recognized in China. Population-based screening programs and education for healthcare providers are urgently needed.
Paul S et al.	Sudan	2019	Misdiagnosis or delayed diagnosis is common in Sudan due to diverse symptoms and limited resources. Cost-effective management strategies and locally sourced gluten-free options are critical.

**b) Infectious conditions of the GI tract**

GI infections present a major health challenge in LMICs, where children face increased exposure to risk factors like inadequate sanitation, malnutrition, poor food hygiene, and limited healthcare infrastructure. Gastroenteritis (GE) is the infection of the stomach and intestines causing acute illness, inflammation, and symptoms such as diarrhea, fever, and anorexia, resulting in pediatric morbidity and mortality. Diarrhea is one of the leading causes of pediatric deaths according to the Global Health Data Exchange in 2016^[30]^. Diarrhea, a common symptom of GE, contributes significantly to pediatric mortality, with an estimated 443,832^[31]^ deaths among children under five and 50,851 deaths among those aged 5–9 globally in 2016, disproportionately impacting LMICs. WHO estimates that diarrhea affects 1.7 billion people annually, making it the third leading cause of death in children aged 1– 59 months^[31]^. Sub-Saharan Africa (SSA) alone reported 290,724 deaths and 371 million cases of childhood diarrhea in 2016, underscoring the urgent need for enhanced healthcare infrastructure and preventive measures in these regions^[32,33]^.

Viruses, particularly rotavirus and norovirus, are the leading causes of GE in children, accounting for over 60% of cases worldwide. Bacterial pathogens like *Campylobacter jejuni, Escherichia coli*, and *Salmonella*, along with protozoa like *Cryptosporidium* and helminths, also contribute significantly to GE, spreading primarily through contaminated food and water, insufficient hygiene, and poor sanitation – common issues in LMICs^[34-36]^.

Vaccination is essential for GE prevention, with the rotavirus vaccine widely implemented, reducing hospitalizations for diarrheal diseases among children under five. As of 2019, 100 countries have the rotavirus vaccine in their regular vaccination schedule; the vaccine has reduced the hospitalization of children under 5 years old due to diarrhea from 38% to 23%^[37]^. Hallowell *et al*. (2020)^[37]^ stated that data measuring the effectiveness of the vaccine in developing countries need to be collected. However, vaccine adoption in LMICs is limited due to challenges in affordability and cold-chain storage, and while vaccines for bacterial causes like cholera and typhoid are available in HICs, they are less accessible in LMICs. Thus, vaccination must be supported by improved hygiene practices and educational efforts to more effectively combat GE in these settings^[37–39]^.

Viral and bacterial gastroenteritis (GE) typically presents with symptoms such as pyrexia, non-bloody diarrhea, vomiting, and abdominal pain, which generally last for 1–2 weeks. If these symptoms persist, the condition can progress to chronic GE^[40,41]^. One of the most concerning outcomes of GE is dehydration, primarily caused by the loss of water and electrolytes through vomiting and diarrhea, which is a leading contributor to mortality in children. Early recognition of GE and dehydration by parents or guardians, coupled with timely medical intervention, is critical for improving treatment outcomes. Public health education plays a crucial role in encouraging caregivers to seek medical attention when symptoms are still in the early stages, thus enhancing the chances of recovery. Treatment typically involves oral and intravenous rehydration fluids, nutritional support (especially for malnourished children), antibiotics for bacterial infections, antiemetic and antidiarrheal medications, and analgesics. In addition to dehydration, GE can result in electrolyte imbalances, metabolic acidosis, food intolerances, hemolytic uremic syndrome (HUS), and an increased susceptibility to further GE infections, which can ultimately lead to death^[42]^. Addressing these challenges is critical to achieving the United Nations’ Sustainable Development Goal (SDG) 3.2, which aims to reduce childhood mortality. Since diarrheal illnesses remain one of the leading causes of pediatric mortality, governments in low- and middle-income countries (LMICs) must prioritize improving public health education, sanitation, food and water infrastructure, and healthcare resources to reduce mortality rates and meet the 2030 target set by the SDGs^[43,44]^.

The economic burden of GE treatment is substantial in LMICs, where treatment costs can range from $213 to $350, often constituting a significant portion of household income, especially when care is sought in private facilities^[45]^. With up to 72% of child hospitalizations in certain regions attributed to GE, the economic strain on families and healthcare systems is considerable^[46]^. The lack of essential facilities, including pediatric endoscopy services and adequate sanitation, further impedes effective GE management. Shortages of antibiotics and clean water exacerbate the vulnerability of children, particularly those already weakened by malnutrition, to severe infections and hinder recovery^[47,48]^. Overburdened healthcare systems in LMICs tend to deprioritize GE care, limiting public health campaigns on prevention and vaccination efforts for rotavirus. This persistent gap in effective GE management results in a high and ongoing burden of disease, contributing to significant morbidity and mortality among children in LMICs^[49]^ (see Table 3).

**Table 3:** Summary of diarrhea and gatroenteritis-focused studies.

Study Author	Country	Date	Major Qualitative Findings
Kotloff KL et al.	LMICs	Jul 2013	Moderate-to-severe diarrhea (MSD) in children under five is a leading cause of morbidity and mortality in sub-Saharan Africa and South Asia. Rotavirus, Shigella, and Cryptosporidium are key pathogens.
Operario DJ et al.	LMICs	Jun 2017	Rotavirus remains the most common cause of severe acute watery diarrhea in children, even in vaccinated regions. Surveillance emphasizes targeting multiple pathogens, including norovirus and adenovirus.
Basharat N et al.	Pakistan	May 2021	Rotavirus is a significant cause of severe diarrhea, leading to high morbidity and healthcare use. Improved vaccination programs and surveillance systems are needed to mitigate outbreaks.

**c) Current state of pediatric GI endoscopy in LMICs**

The increasing burden of pediatric GI diseases in LMICs highlights the urgent need for pediatric upper GI endoscopy, a crucial diagnostic and therapeutic tool. In regions like South Asia and Africa, the incidence of GI diseases in children is growing, with significant health impacts. Conditions such as chronic NSAID-induced gastritis are common in countries like India, where over-the-counter availability of NSAIDs leads to their widespread use and subsequent complications in children^[50]^. Upper GI bleeding (UGIB) is another key indication for endoscopy in LMICs. Hematemesis, resulting from causes like ingested maternal blood, food impaction, or epistaxis, is frequently seen in South Asian and African contexts. A study from Thailand found that over half of the critically ill children in the ICU developed UGIB, emphasizing the vulnerability of pediatric patients to severe GI complications^[51,52]^. Other upper GI conditions, such as peptic ulcers and esophageal abnormalities, including those related to malignancies, are increasingly diagnosed in LMICs like Senegal. These conditions often remain undetected until they have significantly progressed, making timely endoscopic intervention crucial to avoid serious health consequences like malnutrition, delayed growth, and increased morbidity and mortality^[53]^. The broad range of indications for pediatric upper GI endoscopy in these settings includes recurrent abdominal pain, vomiting, dyspepsia, dysphagia, heartburn, portal hypertension, and ingestion of corrosive substances. Endoscopic findings in these cases commonly include gastritis, gastric erosions, esophageal varices, duodenitis, gastric ulcers, and gastric polyps. If untreated, these conditions can lead to severe complications, highlighting the indispensable role of endoscopy in pediatric healthcare in LMICs^[12]^.

Despite the critical need for upper GI endoscopy, expanding its availability and accessibility in LMICs faces substantial challenges. Financial constraints are a primary obstacle, preventing the procurement of essential pediatric endoscopic equipment, such as smaller scopes and child-specific accessories, which are necessary for safe and effective procedures. In regions like Eastern Africa, where the burden of pediatric GI diseases is high, the capacity for pediatric endoscopy is severely limited. The shortage of pediatric endoscopists and appropriate equipment exacerbates the situation, leaving many children without timely diagnostic and therapeutic interventions^[54]^. The scarcity of trained pediatric specialists further hampers the development of pediatric endoscopy services, and many areas struggle with even basic diagnostic endoscopy. More advanced therapeutic procedures are almost non-existent in these regions, making the need for proper training and equipment even more urgent. In some cases, non-physician clinicians and specially trained nurses are tasked with performing pediatric endoscopy due to the shortage of pediatric gastroenterologists. While this approach addresses some immediate needs, it raises concerns about the quality and safety of care provided^[54,55]^.

The prohibitive cost of pediatric endoscopic services also limits access, with many families unable to afford necessary procedures, exacerbating health disparities and delaying diagnoses. This economic barrier contributes to poorer outcomes for children in LMICs. However, efforts to improve pediatric GI care in these regions are underway. Collaborative initiatives between HICs and LMICs have led to some progress, including training programs for local healthcare providers and the provision of essential equipment. Partnerships focused on training local specialists and renovating facilities to accommodate pediatric patients have shown promise. Despite these advancements, much work remains to bridge the gap between the availability of pediatric GI services in LMICs and HICs, highlighting the need for continued efforts to expand pediatric upper GI endoscopy services and address the systemic barriers to care^[54–56]^ (see Table 5).

**d) Pediatric hepatology in LMICs**

Hepatology, the medical field focused on liver diseases, is crucial for global health, especially in LMICs, where liver diseases like viral hepatitis, cirrhosis, and hepatocellular carcinoma are major contributors to morbidity and mortality. These regions often face significant challenges, including limited healthcare infrastructure, lack of vaccinations, and inadequate preventive measures, all of which exacerbate the burden of liver diseases. Pediatric liver diseases in LMICs are predominantly infectious, with hepatitis A (HAV), B (HBV), and E (HEV) being common.

The World Health Organization (WHO) estimates the global burden of hepatitis B and C combined to be 304 million cases. The global incidence of hepatitis B is approximately 1.23 million, with a mortality rate of 1.1 million. In comparison, hepatitis C has an incidence of 98,000 and a mortality rate of 218,000. From 1990 to 2019, the global mortality rates for hepatitis A have generally decreased, except in the Australasia region^[57]^. However, the incidence of hepatitis A has been rising in several low-income regions, as well as in a few high-income regions. In West Africa, a study found that Benin had the highest prevalence of pediatric hepatitis B at 10%, while Togo had the lowest at 1%. This study also highlighted the critical role of vaccination, with vaccinated children exhibiting a significantly lower prevalence (2%) compared to unvaccinated children (6%)^[58]^.

In Pakistan, up to 75% of children have been affected by HAV, while in India, HAV and HEV are endemic, contributing to high pediatric morbidity. HBV remains a leading cause of both acute and chronic liver disease, with high prevalence rates in regions like sub-Saharan Africa, where up to 8% of children under five are affected. Transmission occurs primarily through mother-to-child and close contact, putting young children at high risk. Co-infection with HIV further complicates the burden of HBV, with 7% of HIV-positive individuals also infected with HBV, especially in regions with limited access to antiviral treatments and vaccines, such as for hepatitis C (HCV)^[5,6,58,59]^.

In addition to viral hepatitis, other conditions like amebic liver abscesses and hydatid cysts are prevalent in India, further burdening pediatric healthcare. However, other hepatological diseases, such as Wilson’s disease and metabolic liver conditions, often go untreated due to the lack of specialized care, diagnostic tools, and expensive treatments like penicillamine. Although liver transplantation provides a potential cure for acute liver failure, end-stage liver disease, and certain cancers, it remains underutilized in LMICs due to financial constraints and logistical challenges. In India, while liver transplantation has advanced with survival rates above 90% in some centers, it is largely driven by the private sector, making it inaccessible for many. Living donor liver transplants are more common than deceased donation programs, which remain limited, particularly in northern India. Financial support through crowdfunding and philanthropic efforts has helped many families in LMICs afford these life-saving procedures, though significant barriers remain to wider accessibility^[60-62]^.

The management of pediatric liver diseases in LMICs is further hindered by a shortage of trained pediatric hepatologists and limited resources for diagnosis and treatment. General pediatricians often manage complex liver conditions without specialized training, leading to delayed diagnoses and worse outcomes. In countries like Nigeria, the number of pediatric hepatologists is dwindling, and chronic liver diseases often go untreated until liver transplantation is necessary. However, due to the scarcity of medical resources, expensive antiviral drugs, and high costs of liver transplantation, effective treatment remains a major challenge in many LMICs. Despite these obstacles, collaborative initiatives between high-income and low-income countries, such as training programs and providing essential medical equipment, have shown promise in improving pediatric hepatology care. These efforts, combined with grassroots financial support, are beginning to make liver transplantation more attainable in these regions, offering hope for children with liver diseases in LMICs^[63,64]^ (see Table 4).

**Table 4:** Summary of liver disease-focused studies.

Study Author	Country	Date	Major Qualitative Findings
Shanmugam N et al.	India	Jul 2021	Hepatitis B, cirrhosis, and liver failure are rising among Indian children. Vaccination programs and better healthcare access are essential, especially in rural areas.
Fofana DB et al.	West Africa	Jan 2023	HIV co-infection significantly increases hepatitis B risk in children. Factors include maternal transmission, poor vaccination, and socioeconomic challenges.

**Table 5:** Summary of other GI conditions-focused studies.

Study Author	Country	Date	Major Qualitative Findings
Kocic M et al.	LMICs	Dec 2023	Causes of upper gastrointestinal bleeding (UGIB) vary with age: vitamin K deficiency in neonates, Mallory–Weiss tears in younger children, and erosive gastritis in older children.
Deerojanawong J et al.	Thailand	Jan 2009	UGIB is common in mechanically ventilated children. Risk factors include coagulopathy and GI diseases. Proactive monitoring and stress ulcer prophylaxis are crucial.

### Challenges in pediatric gastroenterology care in developing countries

**Figure 1.**
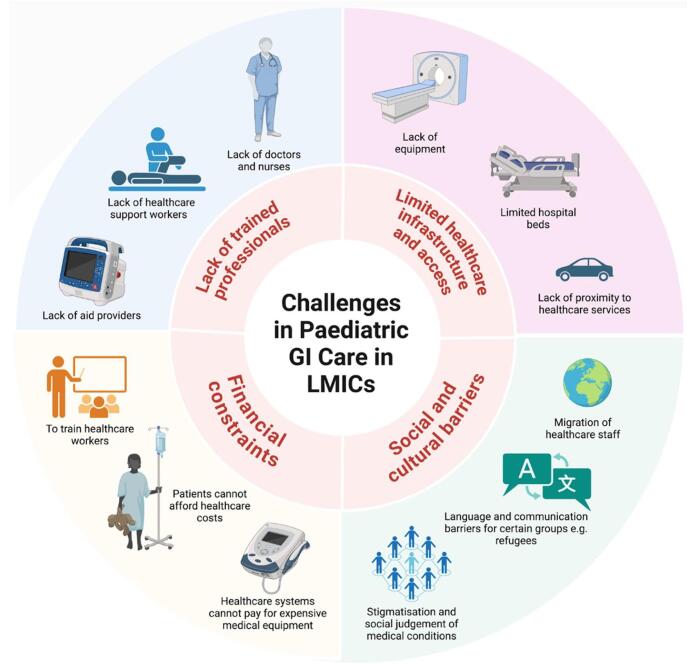
Challenges in pediatric GI care in LMICs.

Pediatric gastroenterology care in developing countries faces numerous challenges, primarily due to limited healthcare infrastructure, resources, and access to specialized services, as referred to in Fig. 1. Many of these regions lack the necessary diagnostic tools and equipment for conditions such as *Helicobacter pylori* infections and chronic liver diseases. Hospitals are often overwhelmed, leading to overcrowding, long waiting times, and overworked healthcare staff, which diminishes the quality of care. Furthermore, geographical barriers, especially in rural areas, exacerbate these problems, as patients are often required to travel long distances to reach healthcare facilities that may not have the required specialists or equipment to effectively manage pediatric gastroenterological conditions, such as severe diarrheal diseases. This results in delayed diagnosis and treatment, increasing the risk of poor health outcomes for children^[5,6]^.

Inadequate sanitation and lack of basic amenities like handwashing facilities in low-income areas also contribute to the high incidence of diarrheal diseases among children. In urban areas like New Delhi, India, poor sanitation has been linked to high rates of diarrheal diseases, which could be reduced by 70% through improved hygiene practices like proper handwashing. This highlights how broader infrastructure issues, such as poor sanitation, worsen conditions like cholera and persistent diarrhea. Additionally, the shortage of healthcare workers, particularly pediatric gastroenterologists, remains a significant issue. Despite efforts such as the African Paediatric Fellowship Programme^[65]^, many countries in sub-Saharan Africa (SSA) and other LMICs face critical shortages of trained specialists. For example, Ethiopia has only about two doctors per 100,000 population, compared to 230 per 100,000 in the UK. The migration of healthcare workers to wealthier countries further exacerbates these shortages, as LMICs lose skilled professionals who are in short supply at home. In countries like Malawi, where 85% of doctors work in urban areas, rural populations face limited access to healthcare, further straining the system^[6,66,67]^.

Economic challenges in LMICs, worsened by the COVID-19 pandemic, have disrupted training programs for pediatric gastroenterology and created gaps in care delivery. Financial limitations hinder the procurement of medical supplies and equipment, while unstable economies make it difficult to retain and train healthcare workers. The lack of funding, compounded by insufficient salaries for medical professionals, prevents many countries from attracting or retaining specialists in fields like pediatric gastroenterology. This issue is particularly acute in countries like Malawi, where only 8% of pediatric gastroenterology posts are filled due to inadequate salaries. Furthermore, many clinical guidelines for managing pediatric gastroenterological conditions, established in developed countries, are not implemented in LMICs due to the high costs of investigations and a lack of insurance. Cultural barriers also play a role in delaying care, as conditions like inflammatory bowel disease (IBD) are often hidden or denied by families due to stigma, preventing timely diagnosis and treatment. Refugees, who often face language and cultural challenges, are particularly vulnerable, as they may be reluctant to seek care due to fear of discrimination or misunderstanding. These factors contribute to the overall healthcare disparity, making access to proper pediatric gastroenterology care even more difficult in LMICs^[5,6,68,69]^.

## Future prospects

### Prevention measures

**Figure 2.**
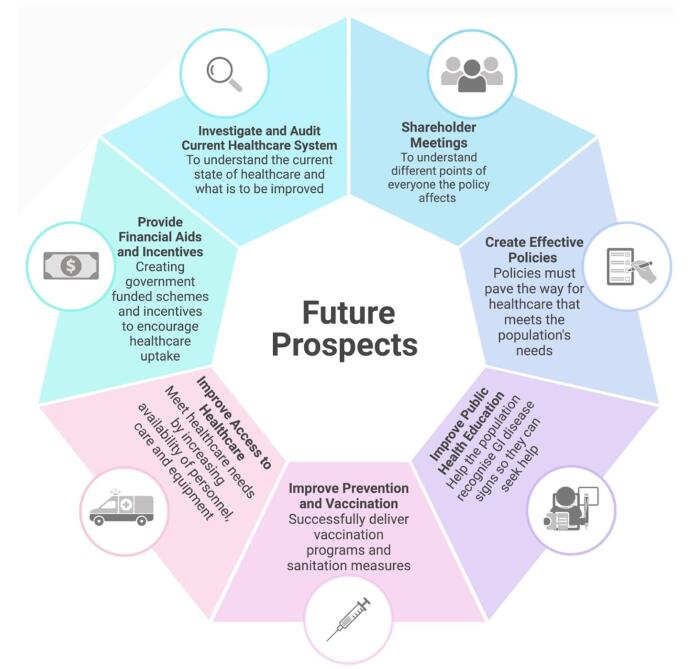
Future prospects and solutions for pediatric GI healthcare in LMICs.

There are many solutions, and future prospects improve pediatric GI care in LMICs (Fig. 2). Public health education is crucial for raising awareness of pediatric gastrointestinal (GI) disorders, as highlighted by Besnier *et al*. (2021)^[70]^. Campaigns focused on infectious GI conditions can inform communities about the risks of contaminated food and water, while preventive strategies like boiling water can mitigate these risks. For chronic conditions such as celiac disease (CeD) and inflammatory bowel diseases (IBDs), educating people to recognize early symptoms ensures timely intervention, potentially preventing complications^[5,6]^. A strong pediatric vaccination program is also essential in reducing the incidence of severe GI diseases, with vaccines for rotavirus, norovirus, hepatitis A (HAV), and hepatitis B (HBV) proving particularly effective. However, challenges in vaccine storage and administration in low- and middle-income countries (LMICs) need to be addressed to improve coverage and reduce wastage^[71]^.

Improving sanitation and access to clean water is vital for preventing pediatric GI disorders, as contaminated water and poor sanitation are major contributors to infections. Efforts should focus on building sanitation infrastructure, promoting hygiene, and ensuring safe drinking water. For example, handwashing stations in schools and community centers can significantly reduce disease transmission^[6,72]^.

### Policy recommendations

To develop effective policies for managing pediatric GI disorders, conducting local assessments such as audits and quality improvement studies is essential to collect data on patient needs and assess whether current practices meet local and global healthcare standards^[73]^. These assessments should be collaborative, involving both government policymakers and local healthcare workers, with support from international organizations like the WHO and UNICEF, especially in resource-limited settings. Key data required for policy development include information on the burden, incidence, and prevalence of pediatric GI diseases, the impact on children and adolescents, the complications, and the effectiveness of current treatments. Understanding the broader public health risks – physiological, social, and psychological – is also critical in creating comprehensive policies^[5,73]^. Additionally, stakeholder engagement is crucial in policy formulation. Involving healthcare providers, government officials, hospital administrators, public health officers, NGOs, and parents helps identify challenges, feasibility issues, and potential solutions. This ensures that policies are effective, aligned with the population’s needs, and culturally appropriate^[5]^. Governments should prioritize funding for preventive and primary healthcare, integrating pediatric GI disorder management into national health strategies, potentially by establishing specialized units within hospitals and clinics, and ensuring that these units are well-resourced^[72]^.

### Widening access to healthcare and investing in staff training

Expanding healthcare access in LMICs requires addressing barriers such as financial constraints, geographic distance, language differences, and limited resources for treating pediatric GI conditions. Strategies should be cost-effective, user-friendly, and tailored to local contexts, such as using battery-operated or solar-powered medical devices in areas with unreliable electricity^[72]^. Bringing healthcare services closer to communities through mobile units or remote visits can also increase service uptake. Additionally, reducing healthcare costs and offering government-funded health schemes can alleviate financial burdens and improve accessibility^[73]^.

A significant challenge is the shortage of healthcare workers in LMICs, which limits care availability. Expanding education and training is critical, yet training programs are often scarce and costly. Subsidized opportunities, such as scholarships for specialized pediatric gastroenterology training, are essential for building local expertise. Programs like those from the World Gastroenterology Organisation (WGO) offer advanced training and send specialists to areas in need of expertise. Furthermore, providing first-aid training in GI-specific emergencies to non-medical personnel, such as parents and teachers, can improve early intervention, reduce mortality rates, and enhance outcomes, particularly in areas with delayed healthcare access^[5,6]^.

## Limitations

A major limitation of this study is the lack of prior research on pediatric gastrointestinal (GI) conditions, particularly non-infectious ones, in low- and middle-income countries (LMICs). The majority of existing data on pediatric conditions, such as inflammatory bowel diseases (IBDs) and celiac disease, primarily originate from high-income countries (HICs), making it challenging to accurately assess the current state of care, incidence, and prevalence of these conditions in LMICs. Many of these countries are located in regions such as Asia, Africa, and South America. To address this gap, future studies must focus on gathering comprehensive data on the prevalence and incidence of pediatric GI conditions in LMICs, in collaboration with international bodies such as the World Health Organization (WHO).

Furthermore, due to the lack of available data from several LMICs, generalizability is a limitation of this review. The findings may not be applicable to all LMICs, as the data included in the literature may not reflect the full diversity of these regions. A potential remedy to this limitation would be to increase research efforts in LMICs and gather more localized data to improve the analysis and support more accurate conclusions.

Another limitation of this review is the aggregation of data from both low- and middle-income countries into a single group. This approach may not adequately represent the current status of pediatric GI diseases and healthcare, as middle-income countries typically have better healthcare infrastructure and lower rates of infectious GI diseases due to advancements in sanitation measures and public health education.

Finally, as this study is a narrative review, the process of selecting studies lacks the rigor of more systematic approaches. To improve upon this, future studies could employ more robust methodologies, such as conducting a systematic review and meta-analysis, utilizing the PRISMA tool for article selection and quality assessment.

## Conclusion

Despite advancements in the diagnosis and management of pediatric gastrointestinal (GI) disorders in low- and middle-income countries (LMICs), the burden of these conditions remains substantial. Persistent challenges, such as limited access to healthcare, a shortage of trained professionals, and inadequate public health infrastructure, continue to impede progress. Poor sanitation, contaminated water, and insufficient public awareness further exacerbate the prevalence of GI diseases among children in these regions. Although targeted interventions – such as vaccination programs, mobile healthcare units, and specialized training – have made some headway, comprehensive, context-specific solutions are still urgently needed. Addressing these barriers through robust policy development, public health education, and significant investments in local healthcare capacity is critical to improving outcomes for paediatric patients.

Going forward, prioritizing preventive measures, including expanding vaccination coverage and enhancing water and sanitation infrastructure, will be vital in reducing the incidence and impact of pediatric GI diseases in LMICs. Strengthening healthcare training programs and fostering collaboration between international organisations, local governments, and community stakeholders will be essential in creating sustainable and equitable healthcare systems, capable of delivering improved care for pediatric populations.

## Data Availability

Data availability does not apply to this article as no new data were created or analysed in this study.

## Research registration unique identifying number (UIN)

Not required.
